# Community health worker interventions are key to optimal infant immunization coverage, evidence from a pretest-posttest experiment in Mwingi, Kenya

**DOI:** 10.11604/pamj.2017.28.21.11255

**Published:** 2017-09-13

**Authors:** Japheth Mativo Nzioki, James Ouma, James Hebert Ombaka, Rosebella Ongutu Onyango

**Affiliations:** 1 Department of Environmental Health, University of Kabianga, Kericho, Kenya; 2 Department of Biomedical Sciences and Technology, Maseno University, Kisumu, Kenya; 3 Department of Public Health, Maseno University, Kisumu, Kenya

**Keywords:** Community health workers, maternal and child health, vaccination coverage

## Abstract

**Introduction:**

Immunization is a powerful and cost-effective health intervention which averts an estimated 2 to 3 million deaths every year. Kenya has a high infant and under five mortality and morbidity rates. Increasing routine child immunization coverage is one way of reducing child morbidity and mortality rates in Kenya. Community Health Workers (CHWs) have emerged as critical human resources for health in developing countries. The Community Strategy (CS) is one of the CHW led interventions promoting Maternal and Child Health (MCH) in Kenya. This study sought to establish the effect of CS on infant vaccination Coverage (IVC) in Mwingi west sub-county; Kenya.

**Methods:**

This was a pretest - posttest experimental study design with 1 pretest and 2 post-test surveys conducted in intervention and control sites. Mwingi west and Mwingi north sub-counties where intervention and control sites respectively. Sample size in each survey was 422 households. Women with a child aged 9-12 months were main respondents.

**Results:**

Intervention site end-term evaluation indicated that; the CS increased IVC by 10.1% (Z =6.0241, P <0.0001), from a suboptimal level of 88.7% at baseline survey to optimal level of 98.8% at end term survey. Infants in intervention site were 2.5 times more likely to receive all recommended immunizations within their first year of life [(crude OR= 2.475, P<0.0001; 95%CI: 1.794-3.414) (adj. OR=2.516, P<0.0001; 95%CI: 1.796-3.5240)].

**Conclusion:**

CS increased IVC in intervention site to optimal level (98.8%). To improve child health outcomes through immunization coverage, Kenya needs to fast-track nationwide implementation of the CS intervention.

## Introduction

Immunization is a powerful and cost-effective health interventions which averts an estimated 2 to 3 million deaths every year [1,2]. World Health Organization (WHO) considers a child to have received all basic vaccinations if he or she has received: BCG vaccination against tuberculosis; three doses of DPT vaccine to prevent diphtheria, pertussis, and tetanus (or three doses of pentavalent, which includes DPT and vaccinations against both hepatitis B and *haemophilus influenza* type B); at least three doses of polio vaccine; and one dose of measles vaccine [2]. These vaccinations should be received during the first year of life 2]. In Kenya, an infant is considered to be fully vaccinated if the infant has received all WHO basic vaccinations and three doses of pneumococcal vaccine [3].

Global vaccination coverage - the proportion of the world's children who receive recommended vaccines - has remained steady for the past few years [1] . Since 2000, Global Alliance for Vaccines and Immunization (GAVI) and the Vaccine Alliance has supported vaccination of 500 million children in the world's poorest countries, saving an estimated 7 million lives [1]. Global routine vaccination coverage by 2014 was 86% - up from 20% in 1980 [1]. The world is closer to realizing a world free from polio. In 1988 there were 350,000 cases of polio in the world and in 2014, only 359 [1]. Measles vaccines have averted an estimated 17.1 million deaths between 2000 and 2014. Maternal and neonatal tetanus has been eliminated from 35 out of 59 high-risk countries since 1999. By the end of 2014, over 47 million children were protected from pneumonia by a pneumococcal vaccine program implemented in 73 countries [1].

A fact sheet [4] reporting on global child immunization coverage indicates the following; In 2014; an estimated 86 % of infants worldwide received DTP3 vaccine - up from 20% in 1980, global coverage of 3 doses of *Haemophilus influenza (Hib)* vaccine was estimated at 64%, and global coverage with 3 doses of *hepatitis B*vaccine was estimated at 83%. By the end of 2015, 85% of children had received 1 dose of measles vaccine by their second birthday, 160 countries had included a second dose as part of routine immunization and 61% of children received 2 doses of measles vaccine. In 2015, 86% of infants around the world received 3 doses of polio vaccine and global coverage of *Pneumococcal vaccine* was estimated at 37%. *Mumps vaccine* had been introduced nationwide in 121 countries by the end of 2015. Further the WHO report indicated that *Rotavirus vaccine* and *Rubella vaccine* global coverages were estimated at 23% and 46% respectively.

In Kenya, infant vaccination Coverage (IVC) (the proportion of children aged 1 year and below who have received the basic WHO recommended vaccines plus three doses of pneumococcal vaccination within their first year of life) is not documented in the most recent KDHS. However, child routine vaccination coverage- the proportion of children aged 12-24 months who have received the basic WHO recommended vaccines plus three doses of pneumococcal vaccination) is at 67.5% [3]. This coverage is low compared to 2015 global routine child immunization coverage of 86% [1]. This could be the cause of high infant mortality rate reported as 39 deaths per 1,000 live births and high under five mortality rates (52 deaths per 1,000 births) by 2014 KDHS [3]. In Kitui county where Mwingi west sub-county is located, Routine child immunization coverage is 52.7%; even lower than the national coverage [3]. These statistics point out to one thing; the need to design and implement innovative interventions to help increase child routine immunization coverage in Kenya. This in turn could help in reducing the high infant and under five mortality rates in the country.

Community health workers (CHWs) have emerged as critical human resources able to extend health systems and basic services directly to communities and households [5]. In resource poor countries, CHWs are increasingly recognized as a critical link in improving access to health services [5]. Evidence from a review of 34 studies from low and middle income countries and other studies in Nepal, Bangladesh, Nigeria, Mali and India has left no doubt that CHW interventions have improved Maternal and Child Health (MCH) outcomes in developing world [6–12]. Community Strategy (CS) also commonly referred to as Community Health Strategy (CHS) is a CHW led Primary Health Care (PHC) intervention which was designed in 2006 to support the delivery of Kenya Essential Package for Health at level one (community level) [13]. In Mwingi west sub county, the intervention was initiated by the Ministry of Public Health and Sanitation (MoPHS) in partnership with the African Medical and Research Foundation (AMREF) in March 2011 as a component of Aphia plus *Kamili* project [14].

As evident in background information, infant and under-five mortality rates are high in Kenya. Routine child immunization coverage which plays a critical role in reducing child mortality rates in the country is low. Though studies have shown that CHWs play an important role in increasing access to health care services, the effect of the CS on infant immunization coverage in Mwingi west sub-county remains unknown. The purpose of this study was to fill this knowledge gap. The study sought to establish the effect of CS on IVC in Mwingi west sub-county. The null hypothesis of this study was ‘In the intervention site, there is no difference in the odds of infants who received all Government of Kenya (GoK) recommended vaccines within 9-12 months of life at baseline survey compared to end-term survey'.

## Methods

### The study area

This was an experimental study with intervention and control site. The intervention site was Mwingi West sub-county and the control site was Mwingi North sub-county. Both sub counties are located in Kitui County. Mwingi West sub-county had a total population of 103,774 people in the 2009 population census with a projection of 111,346 people by 2015. While Mwingi north sub county was reported to have a total population of 139,967 in 2009 population census with a projection of 150,179 persons by 2015 [15]. The intervention and control sites have similar climatic and ecological characteristics, poor infrastructure and are located in a rural arid and semiarid area [16].

### The intervention

CS is a CHW led intervention with the following key elements;

***Community mobilization:*** MoPHS and AMREF-Kenya mobilized community in Mwingi west sub-county through community meetings led by local chiefs (popularly referred to as chief barazas). The aim was to create awareness of the new intervention and mobilize community members to select potential volunteer CHWs for training.

***Identification and training of volunteer CHWs:*** Identified volunteer CHWs were trained on PHC service provision and formation and maintenance of Community Units (CUs).

***Enumeration, mapping of households and creating Community Units (CUs):*** Enumeration of the community members was conducted at household level. The product of this exercise was household registers with demographic data of households. A total of 10 CUs were created namely; *Kisovo, Waita, Kyethani, Kairungu, Nzeluni, Kea, Kalanga, Mutyangome, Munyuni, and Wikithuki* CUs.

**Recruitment and training of Community Health Extension Workers (CHEWs):** CHEWs were selected from medical staff trained at certificate and/or diploma levels and working for the Ministry of Health. These professionals were identified from dispensaries and health centers within the CUs, trained and recruited to work in the CS intervention. Their role was to support, supervise and coordinate CHWs with each CHEW supervising up to 25 CHWs. CHEWs also facilitated health education meeting sessions in the community and provided a linkage between CHWs and health facilities.

***Health service provision:*** The responsibility of CHWs was to provide day to day health services at household level. These services included; promotion of community hygiene and environmental sanitation, provision of Insect Treated Mosquito Nets (ITNs), child immunization services, provision of essential drugs, health education and counselling. Other MCH services provided by CHWs include; provision of family planning services, identification and tracking of newly expectant women to ensure that; they seek ANC services as recommended, they delivered under care of skilled medical professionals, they went through postnatal care, and that their infants received vaccines in Routine Child Immunization Program (RCIP) in time. CHWs also played a role in detecting complications related to pregnancy and child birth and providing referral services for treatment at dispensaries and health centers. CHWs further monitored the health of newborn babies within their CUs and provided referrals for any sick child for treatment at the local health centers. Study participants in the control site received standard MCH care provided by the Government of Kenya (GoK).

### The research design

This was a non-randomized prospective experimental study in which 1 pre- test and 2 post-test time series household surveys were conducted in both intervention and control sites. Data was collected at 3 time points; a baseline survey was used to collect baseline data in both intervention site and control sites. First post intervention survey data was collected 9 months after implementation of the CS in intervention site and control site. This survey was defined as mid-term evaluation. Second post intervention survey data was collected in both intervention and control sites 18 months after implementation of the CS. This is defined as end-term evaluation survey. Data was collected at household level with women of reproductive age with a child aged 9-12 months being the main respondents. Based on nature of phenomena to be examined, data was collected from different participants in all the three surveys. For example, it was not possible to guarantee that a woman who was sampled at baseline survey and data collected on her quality of ANC services provided, place of delivery etc., will be expectant again after 9 months or even 18 months to enable investigators to measure the same parameters again. This informed the choice of having different participants at baseline, midterm and end-term evaluation surveys.

### Sample size determination

Reference [17] provides the Fisher's formula for calculating a representative sample size of a population with more than 10,000 participants. After employing this formula, a representative sample size of 384 households was established. Thirty-eight households (10 percent of 384 households) were added into this sample in order to carter for non-response. A total sample size of 422 households was determined.

### Sampling procedure

Purposive and simple random sampling methods were employed. Purposive sampling was used to identify intervention and control sites. Mwingi west Sub County was purposively selected as intervention site based on the fact that the CS program was to be implemented in the sub county. Mwingi north sub county was also purposively sampled as the control site based on the following; CS was not under implementation in the sub county, the sub county borders Mwingi West and both sub-counties have many similarities which include similar ecological and climatic characteristics [15].

Simple random sampling was applied in all the pre-and post-intervention surveys in the study and control sites. The first step was to develop a sampling frame for each of the three surveys conducted in the study site and the control site respectively. Sampling frames in Mwingi west Sub County was 1243 households (in Waita CU) at baseline and 927 households (in Kyethani CU) and 1107 households (*Wikithuki* CU) at midterm and end term surveys respectively. The sampling frame was developed using household registers which were developed during creation of CUs. In the control site, the researchers together with village elders and local chiefs conducted a series of community meetings to help in identification of households with a child or children aged between 9-12 months. This was done *in Kyuso, Ngomeni* and *Mumoni* wards. A sampling frame of 971 households, 1032 households and 1208 households was developed in *Kyuso, Ngomeni* and *Mumoni* wards respectively. Using SPSS a sample size of 422 households was drawn from each sampling frame.

### Data collection process

The first step in data collection was to conduct a pre-intervention survey to collect baseline data in both intervention and control sites. The aim was to obtain pretest measurements on both intervention and control groups to allow assessment of initial comparability of the two groups. In the intervention site, baseline data was collected from a total 416 households in Waita Community CU while in the control site baseline data was also collected from a total of 411 households in control site. This exercise took place from March 2012 to June 2012. Baseline survey was followed by two post intervention surveys in both intervention and control sites. Data for first post intervention survey (mid-term survey) was conducted 9 months (from March 2013 to June 2013) after implementation of the CS in Mwingi west Sub County. In the intervention site data was collected in 413 households in Kyethani CU while in the control site data was also collected from 413 households. The second post intervention survey took place 18 months (from March 2014 to June 2014) after implementation of the CS. In this survey, data collection in intervention site was done from 417 households in Wikithuki CU and in the control site data was collected from 420 households.

### Variables in the study

The independent variable is the study was CS intervention while the dependent variable was infant vaccination Coverage (IVC). IVC in this study was defined as ‘the proportion of children aged 1 year and below who had received all the basic WHO recommended vaccines plus three doses of pneumococcal vaccination within their first year of life as recommended by the GoK'. In Kenya, a 1 year old child is considered to have been fully vaccinated if they have received the following vaccines; Bacillus Calmette-Guérin -BCG (at birth), Oral Polio Vaccine-OPV (at birth, 6wk, 10wk and 14wk), Pentavalent vaccine-(containing: Diphtheria, Pertussis, and Tetanus (DPT) and vaccinations against both hepatitis B and haemophilus influenza type B)) (given at 6wk, 10wk and 14wk), Pneumococcal vaccine-PCV 10 (administered in 6wk, 10wk and 14wk), and Measles (first dose administered at 9 months). The outcome variable in this study was change in IVC. It was measured in two ways: one; change in the proportion of infants in Mwingi west sub-county who were fully vaccinated by the age of 1 year and two; change in probability that an Infant would be fully vaccinated before and after the intervention.

### Study validity and reliability

A pilot study was conducted in Nzeluni in Mwingi west sub-county before the main study. The objective of the pilot was to test the reliability of data collection tool. Data was collected in a randomly selected sample of 45 households (slightly above 10 per cent of the sample size) in three villages in Nzeluni sub location. Upon testing the data on reliability, the coefficient of internal consistency (Cronbach's *alpa*) was 0.864. This value was within the recommended range of 0.70-0.95 [18] and therefore we were assured that the data collection tool (questionnaire) was reliable. Internal validity of the study was ensured by applying a sound methodology while external validity was ensured by use of a representative sample size.

### Data analysis and presentation

Frequencies and percentages were used to provide descriptive statistics in this study. Z score tests were used to determine if proportions of IVC before and after the intervention were significantly different. Binary logistic regression was used to control for potential confounders (socio-demographic characteristics) and to establish the odds of infants who had received all recommended vaccines within 1 year before and after the intervention. Data was presented using tables.

### Study limitations

The study had several important limitations; the most important of these was selection of intervention and control sites. Since implementation of the CS was a MoPHS and AMREF-Kenya project which was designed to be implemented in Mwingi West sub county as a whole, it was not feasible to randomly assign the CS intervention to community members in Mwingi west sub county. This is the reason why a non-randomized pre-test and post-test experimental study design was deemed appropriate. Though this method has been employed in other similar studies [8,19–22] the design is weaker compared to a randomized controlled trial. Secondly, researchers were also not able to account for possibility of other programs that could influence MCH outcomes of interest in the intervention site. However, there was an attempt to reduce the effect of confounding factors through, treating socio-demographic characteristics of both intervention and control sties as potential confounders and having them controlled in the binary logistic regression model used in data analysis, and by matching the control to the intervention sites by geographical location, and infrastructural characteristics.

Part of data collection involved collecting data from a Mother and Child Health (MCH) booklet at the household level. In the event that this booklet was not available, respondents were requested to remember the MCH events that happened in a span of 12 months. Though this method has been successfully used in other studies including Demographic and Health Surveys (DHS) [3], the method introduced a retrospective data collection aspect that required respondents to recall past events. Though this was limited only to respondents who could not produce their mother and child booklets, it was a potential source of recall bias error.

### Ethical considerations

Ethical clearance for this study was provided by the National Council of Science and Technology (NCST) of the Government of Kenya (GoK).

## Results

### Socio-demographic characteristics of respondents

Data on socio-demographic characteristics of respondents of this study is summarized in Table 1.

**Table 1:** Socio demographic characteristics of respondents

Variable	Categories	Baseline Survey				Midterm Survey (9 Months)				End term Survey (18 months)			
		Mwingi West		Mwingi North		Mwingi West		Mwingi North		Mwingi West		Mwingi North	
Age		F	%	F	%	F	%	F	%	F	%	F	%
	16-20 years	8	1.9	12	2.9	14	3.4	18	4.4	29	7.0	20	4.8
	21-25 years	35	8.4	63	15.3	61	14.8	59	14.3	64	15.3	76	18.1
	26-30 years	106	25.5	134	32.6	141	34.1	127	30.8	112	26.9	117	27.9
	31-35 years	149	35.8	139	33.8	126	30.5	143	34.6	132	31.7	138	32.9
	36-40 years	113	27.2	57	13.9	69	16.7	59	14.3	80	19.2	63	15.0
	41-45 years	5	1.2	6	1.5	2	0.5	7	1.7	0	0	6	1.4
Parity	1 Child	20	4.8	23	5.6	25	6.1	22	5.3	13	3.1	30	7.1
	2 children	19	4.6	22	5.4	28	6.8	15	3.6	26	6.2	13	3.1
	3 children	60	14.4	58	14.1	74	17.9	64	15.5	65	15.6	67	16.0
	4 children	105	25.2	124	30.2	93	22.5	93	22.5	122	29.3	89	21.2
	5 children	93	22.4	89	21.7	95	23.0	113	27.4	99	23.7	100	23.8
	6 children	63	15.1	74	18.0	66	16.0	82	19.9	65	15.6	88	21.0
	6 and above	56	13.5	21	5.1	32	7.7	24	5.8	27	6.5	33	7.9
Education Level	No education	33	7.9	12	2.9	25	6.1	16	3.9	27	6.5	8	1.9
	Primary level	141	33.9	86	20.9	127	30.8	108	26.2	102	24.5	124	29.5
	Secondary level	149	35.8	228	55.5	167	40.4	187	45.3	208	49.9	167	39.8
	College/University	93	22.4	85	20.7	94	22.8	102	24.7	80	19.2	121	28.8
Occupation	Not working	8	1.9	10	2.4	13	3.1	15	3.6	34	8.2	15	3.6
	Peasant Farmer	206	49.5	233	56.7	225	54.5	247	59.8	226	54.2	230	54.8
	Business	105	25.2	117	28.5	91	22.0	92	22.3	99	23.7	108	25.7
	employment	97	23.3	51	12.4	84	20.3	59	14.3	58	13.9	67	16.0
Marital Status	Single	21	5.0	31	7.5	30	7.3	44	10.7	40	9.6	34	8.1
	Married	306	73.6	350	85.2	299	72.4	328	79.4	311	74.6	337	80.2
	Windowed	24	5.8	12	2.9	16	3.9	15	3.6	18	4.3	18	4.3
	Sep./ Divorced	65	15.6	18	4.4	68	16.5	26	6.3	48	11.5	31	7.4
Monthly Income	≤2500	118	28.4	219	53.3	153	37.0	221	53.5	161	38.6	242	57.6
	2501 - 5000	129	31.0	109	26.5	122	29.5	94	22.8	133	31.9	86	20.5
	5001 - 7500	45	10.8	32	7.8	53	12.8	29	7.0	47	11.3	22	5.2
	7501 - 10000	66	15.9	12	2.9	14	3.4	18	4.4	15	3.6	19	4.5
	> 10000	58	13.9	39	9.5	71	17.2	51	12.3	61	14.6	51	12.1
F. and %. totals each Variable		416	100	411	100	413	100	413	100	417	100	420	100

### Routine child immunization coverage for specific vaccines, intervention site vs control site

*BCG* vaccination coverage in intervention site was; 93.5%, 97.8% and 98.6% at baseline, midterm and end term surveys respectively and 92.2%, 87.4%, and 94.3% at baseline, midterm and end term surveys in control site respectively. *Oral Polio* vaccination coverage (4 doses of polio) in intervention site was, 94.7%, 96.9% and 98.8% at baseline, midterm and end term surveys respectively and 92.2%, 91.3% and 92.6% at baseline, midterm and end term surveys in control site respectively. *Penta valent* vaccine (which includes 3 doses of DPT and vaccinations against both *hepatitis B* and *haemophilus influenza* type B) was; 95.4%, 97.6% and 98.8% at baseline, midterm, and end term surveys respectively in intervention site and 92.7%., 88.1%, and 90.2% at baseline, midterm and end term surveys respectively in control site. *Pneumococcal vaccination* (3 doses) coverage in intervention site was; 91.1%, 96.9% and 98.8% at baseline, midterm and end term surveys respectively and; 90%, 85.4% and 90.5% at baseline, midterm and end term surveys in control site respectively. *Measles vaccination* coverage in intervention site was; 89.7%, 94.7%, and 98.8%, at baseline, midterm and end term surveys respectively and; 86.6%, 84%, and 87.4% at baseline, midterm and end term surveys in control site respectively. These results are summarized in Table 2.

**Table 2:** Routine child immunization coverage of specific vaccines in intervention and control sites

No.	Vaccine	Intervention site						Control Site					
		Baseline		Midterm		End term		Baseline		Midterm		End term	
		Frq.	%	Frq.	%	Frq.	%	Frq.	%	Frq.	%	Frq.	%
1	BCG Immunization	389	93.5	404	97.8	411	98.6	379	92.2	361	87.4	396	94.3
2	Oral polio (4 doses)	394	94.7	400	96.9	412	98.8	379	92.2	377	91.3	389	92.6
3	Penta valent (3 doses)	393	94.5	403	97.6	412	98.8	381	92.7	364	88.1	379	90.2
4	Pneumococcal (3 doses)	379	91.1	400	96.9	412	98.8	370	90.0	349	84.5	380	90.5
5	Measles (1 dose)	373	89.7	391	94.7	412	98.8	356	86.6	347	84	367	87.4

Base line: conducted before intervention

Midterm survey: conducted 6 months after CS intervention

End Term Survey: conducted 18 months after CS intervention

### Infant vaccination Coverage (IVC) in intervention site and control site

IVC: defined as the proportion of children aged 1 year and below who have received the basic WHO recommended vaccines plus three doses of pneumococcal vaccination [2] was as follows; 87.7%, 92.5%, and 98.8%, at baseline, midterm and end-term surveys in intervention site respectively and 84.4%, 83.3%, and 86% at baseline, midterm and end term surveys in control site respectively. These results are summarized in Table 3.

**Table 3:** Z score tests measuring difference in proportions of infant immunization coverage

Study site	Baseline	Mid-term	End term	Midterm Vs Baseline	End term VsBaseline
Intervention	88.7% (369/416)	92.5% (382/413)	98.8% (412/417)	Z =1.8698 P =0.06148	Z =6.0241 P <0.0001^+^
Control	84.4% (347/411)	83.3% (344/413)	86.0% (361/420)	Z =--0.4429. P =0.65994	Z =0.6186 P = 0.53526
Int *Vs* Ctr	Z = 1.8026 P =0.07186	Z = 4.0533 P <0.0001^+^	Z score= 6.9941. P <0.0001^+^		

Base line: conducted before intervention

Midterm survey: conducted 6 months after CS intervention

End Term Survey: conducted 18 months after CS intervention

### Effect of CS on IVC

Effect of CS intervention on IVC was estimated in two ways: one; by measuring if there is a significant difference between IVC before and after CS intervention in both intervention and control group, and two; by comparing the odds of infants who had received all recommended vaccines within 1 year before and after CS intervention in both intervention and control group.

### Z score tests estimating if proportions of IVC before and after CS intervention are significantly different

Initial comparability of intervention and control sites at baseline indicates a difference of 4.3% in IVC (88.7% -84.4%). A Z score test revealed no significant difference between the 2 proportions (Z = 1.8026: P >0.05). In the intervention site, IVC at baseline increased by 3.8% at midterm (92.5%-88.7%) and by 10.1% at end term (98.8%-88.7%). Z score tests indicated no significance difference between midterm survey IVC and baseline survey IVC (Z=1.8698: P>0.05) but the proportion of IVC at baseline was found to be significantly different with proportion of IVC at end term (Z =6.0241: P <0.0001). In the control site, difference between midterm survey IVC and baseline survey IVC was -1.1% (83.3%-84.4%) while the difference between end term survey IVC and baseline survey IVC was 2.7% (86%-83.3%). Z score tests indicated no significance difference between; midterm IVC and baseline IVC (Z =-0.4429: P >0.05) and baseline IVC and end term IVC (Z=0.6186: P=>0.5). These results are summarized in Table 3.

### Odds of receiving recommended vaccines among infants before and after CS intervention in intervention and control sites

We measured both crude and adjusted odds of an infant receiving all the recommended immunizations within 1 year of age before and after the CS intervention in both intervention and control sites. In the adjusted odds ratios; age, parity, maternal education, income and marital status were controlled as potential confounders in the binary logistic regression analysis. In intervention site, no significant difference in the odds of an infant receiving all recommended vaccines were observed in the midterm survey compared to baseline survey ((crude OR=1.570, P>0.05; 95% CI: 0.976-2.525) Adj. OR=1.571, P>0.05; 95%CI: 0.960-2.572)). However, a significant difference in the in the odds of an infant receiving all recommended vaccines was observed between baseline survey and end term survey. In both the crude and adjusted odds ratios infants in the end term survey were 2.5 times more likely to receive all recommended immunizations in the RCIP compared to infants at baseline ((crude OR=2.475, P<0.0001; 95% CI: 1.794-3.414) *Adj.* OR=2.516, P<0.0001; 95%CI: 1.796-3.524)).

In the control site, no significant difference in the odds of infants receiving all recommended immunizations within their first year of life was observed between baseline and midterm surveys and between baseline and end term surveys respectively. The odds ratios of the comparison between midterm survey and baseline survey and baseline survey and end term survey in control site are; (crude OR=0. 9^20^, P>0.05; 95% CI: 0.634-1.333) Adj. OR=0. 942, P>0.05; 95%CI: .643-1.379)] and [(crude OR=1.061, P>0.05; 95% CI: 0.879-1.280) Adj. OR=1.089, P>0.05; 95%CI: 0.898-1.321)] respectively. These results are summarized in Table 4.

**Table 4:** Odds of receiving all recommended vaccines among infants before and after CS intervention in intervention and control sites

			Sig	OR	95%CI
Intervention site	Midterm vs Baseline	Crude OR	0.063	1.570	0.976-2.525
		Adjusted OR	0.072	1.571	0.960-2.572
	End term Vs Baseline	Crude OR	0.0001^+^	2.475	1.794-3.414
		Adjusted OR	0.0001^+^	2.516	1.796-3.524
Control Site	Midterm vs Baseline	Crude OR	0.658	0. 920	0.634-1.333
		Adjusted OR	0. 757	0. 942	0.643-1.379
	End term Vs Baseline	Crude OR	0. 540	1.061	0.879-1.280
		Adjusted OR	0. .384	1.089	0.898-1.321

### Hypothesis testing

The null hypothesis in this study was; ‘In the intervention arm, there was no difference in the odds of infants who received all GoK recommended vaccines within 9 to 12 months of life at baseline survey compared to end-term survey'.

As shown in section 3.4.2., binary logistic regression established a significant difference in the in the odds of an infant receiving all GoK recommended vaccines within 9 to 12 months between baseline survey and end-term survey. In both the crude and adjusted odds ratios, infants in the end term survey were 2.5 times more likely to receive all GoK recommended immunizations in the routine child immunization program compared to infants at baseline ((crude OR=2.475, P<0.0001; 95% CI: 1.794-3.414) Adj. OR=2.516, P<0.0001; 95%CI: 1.796-3.524)). (Hypothesis test statistic is in Table 4.

Based on this test, the null hypothesis was rejected and the alternative hypothesis (In the intervention arm, there was a significant difference in the odds of infants who received all GoK recommended vaccines within 9-12 months of life at baseline survey compared to end-term survey) was adopted.

## Discussion

### Effect of CS on immunization coverage of specific vaccines in the RCIP

This data reveals that coverage of vaccines recommended in RCIP improved in intervention site compared to control. As shown in Table 2, BCG, Penta valent, polio, pneumococcal and measles immunization coverages gradually increased from baseline survey to end-term survey in the intervention site. Control site paints a completely different picture on the same immunization coverages; data indicates a slight drop of immunization coverage between baseline and midterm and another slight increment in immunization coverages between baseline and end-term surveys. These observations could be explained by the following: in the intervention site; the gradual increase in the specific coverages could be as a result of the effect of the intervention. This is based on the fact that at midterm survey, CS intervention had been implemented in the intervention site for a period of six months. This could be the cause of the gradual increment of vaccination coverages from baseline to midterm survey. At end-term survey CS had been implemented in intervention site for 18 months. This again could account for the increment of immunization coverages from midterm to end-term. CHWs in the intervention site could have been highly effective in tracing women with infants in their CUs and ensuring that they did not delay or miss their infant immunization schedules. This could explain the observed increment on immunization coverages. These results further point out that effectiveness of CHWs in following up mothers with infants to ensure that they received recommended immunizations could have improved over time. This could be the reason why end-term survey posted the highest immunization coverages in intervention site. In the control site, two things could account for the slight drop of immunization coverages at midterm compared to baseline; one, the control site did not benefit from the CS intervention and this could be the reason why no increase at midterm evaluation in control site was observed as compared to intervention site. The second thing which could have caused the slight drop in immunization coverage at midterm compared to baseline could be that midterm survey site in control group had more pockets of *the kavonokya* religious group compared to baseline survey site in the same group. Previous studies have shown that members of the *kavonokya* group in Kitui county have been found to shun MCH service utilization including immunization services [23]. As shown in Table 2, the slight increment in immunization coverage of specific immunizations at end-term survey compared to midterm survey in control site was within range with baseline immunization coverages in the same site. Though no tests were done to show if proportions in specific immunization coverages were significantly different in intervention and control site, data indicates that in intervention site, end term survey immunization coverages were all at optimal levels compared to control site. This could only be attributed to the effect of CS intervention.

### Effect of CS on IVC

As shown in Table 3, IVC in intervention site increased from baseline survey to midterm survey and from baseline survey to end-term survey by 3.8% and by 10.1% respectively. Z score tests revealed no significant difference in baseline IVC and midterm IVC proportions and a significant difference between baseline IVC and end-term IVC proportions (Z =6.0241; P <0.0001). In control site, midterm survey IVC decreased by 1.1% compared to baseline survey IVC. In the same site end-term IVC increased by 1.6% compared to baseline survey IVC. As shown in Table 3, Z score tests revealed no significant difference between midterm IVC and baseline IVC as well as between end-term IVC and baseline IVC. Initial assessment of IVC at baseline did not indicate a significant difference between baseline IVC in intervention site (88.7%) and baseline IVC (84.4%) in control site (Z = 1.8026 P>0.05).

This observation can be attributed to the effect of the CS in the intervention site. As explained previously, CHWs in the intervention site could have been highly effective in tracing women with infants in their CUs and ensuring that they did not delay or miss their infant immunization schedules. Z score tests indicate increased IVC at midterm survey in intervention site was not significantly different from IVC at baseline and that IVC at end-term was significantly different from IVC at baseline. This further strengthens the argument that effectiveness of CHWs in tracing mothers with infants and ensuring that their infants received all recommended vaccinations within their first year of life improved overtime. The lack of significant increase in IVC at baseline, midterm and end-term survey in control site could only be explained by the absence of the intervention in control site.

This argument is further supported by a binary logistic which controlled maternal age, parity, maternal education, household income and marital status as confounding factors. In this model infants in the intervention site were found to be 2.5 times more likely to have received all the recommended vaccines within 1 year of age in the end-term survey compared to baseline survey ((crude OR= 2.475, P<0.0001; 95%CI: 1.794-3.414) (adj. OR=2.516, P<0.0001; 95%CI: 1.796-3.5240)). No significant difference in the odds of infants receiving recommended vaccines within their first year of age was observed between baseline and midterm surveys in intervention site. In the control site, there was also no significant difference in the odds of infants receiving recommended vaccines within their first year of age between baseline and midterm surveys, and between baseline and end-term surveys.

Findings in this study are consisted with other studies conducted in resource poor countries. Recent studies have associated increased community participation in immunization with improvements in vaccination coverage [24]. A WHO report on global experience of CHWs in delivery of health-related Millennium Development Goals (MDGs) indicates that CHWs played a critical role in improving MCH outcomes in the last decade through improving child immunization coverage in resource poor countries. CHWs role according to WHO was tracking child immunization defaulters and referring them to health facilities [25]. A review of 17 studies conducted in 10 developing countries reported that CHWs were effective in promoting child immunization coverage [26]. In Kenya a study conducted to evaluate effectiveness of the CS in promoting positive health outcomes also reported that CS was effective in improving measles immunization coverage [18]. These studies support that the considerable increase in IVC in the intervention site was the effect of CHWs working in the CS intervention and not by chance.

## Conclusion

In Mwingi west sub county (intervention site), IVC improved from suboptimal level (88.7%) to optimal level (98.8%). Though the study deign limitations reduces the strength of evidence in this study, it is highly probable that the observed increase in IVC in intervention site was due to effect of CS in the site. To improve child health outcomes through immunization coverage, Kenya needs to fast-track nationwide implementation of the CS intervention.

### What is known about this topic

- Immunization is one of the most powerful and cost-effective of all health interventions and averts an estimated 2 to 3 million deaths every year;
- CHW led interventions have been associated with improved MCH outcomes.

### What this study adds

- This study provides evidence proofing that a CHW led Primary Health Care (PHC) intervention in Kenya increased infant vaccination coverage to optimal levels.

## Competing interests

The authors declare no competing interests.
